# Thin Film Differential Photosensor for Reduction of Temperature Effects in Lab-on-Chip Applications

**DOI:** 10.3390/s16020267

**Published:** 2016-02-20

**Authors:** Giampiero de Cesare, Matteo Carpentiero, Augusto Nascetti, Domenico Caputo

**Affiliations:** 1 Department of Information Engineering, Electronics and Telecommunications, University of Rome, via Eudossiana 18, 00184 Rome, Italy; matteo.carpentiero@gmail.com (M.C.); domenico.caputo@uniroma1.it (D.C.); 2 School of Aerospace Engineering, Sapienza University of Rome, via Salaria 851/881, 00138 Rome, Italy; augusto.nascetti@uniroma1.it

**Keywords:** amorphous silicon sensors, lab-on-chip, differential photodiodes

## Abstract

This paper presents a thin film structure suitable for low-level radiation measurements in lab-on-chip systems that are subject to thermal treatments of the analyte and/or to large temperature variations. The device is the series connection of two amorphous silicon/amorphous silicon carbide heterojunctions designed to perform differential current measurements. The two diodes experience the same temperature, while only one is exposed to the incident radiation. Under these conditions, temperature and light are the common and differential mode signals, respectively. A proper electrical connection reads the differential current of the two diodes (ideally the photocurrent) as the output signal. The experimental characterization shows the benefits of the differential structure in minimizing the temperature effects with respect to a single diode operation. In particular, when the temperature varies from 23 to 50 °C, the proposed device shows a common mode rejection ratio up to 24 dB and reduces of a factor of three the error in detecting very low-intensity light signals.

## 1. Introduction

Lab-on-Chip (LoC) systems have seen remarkable development in the fields of biology and chemistry, because they can perform biochemical analyses with smaller volumes of reagent and in shorter times than standard methods [1]. Optical detection of biomolecules is characterized by a very high sensitivity [2], and for this reason, it is one of the most used techniques in LoC devices. In labeled approaches, recognition is related to the detection of the light produced by fluorescent markers attached to the molecules, while in label free methods, bioassay analysis relies on the measurement of molecule absorbance in the ultraviolet (UV) range or on the detection of bio/chemiluminescent signals.

Recently, on-chip detection methods, based on thin film organic and inorganic sensors have been proposed [3,4,5] as substitutes of cooled charge coupled devices or complementary-metal-oxide-semiconductor imagers, to allow the fabrication of more compact LoC devices with high sensitivity performance.

In particular, several research groups have developed hydrogenated amorphous silicon (a-Si:H) photodiodes to perform both labeled and label free analysis [6,7,8,9,10]. The basic structure of the photosensors is an amorphous silicon carbide (a-SiC:H) p-doped/intrinsic a-Si:H/n-doped a-Si:H stack deposited by Plasma Enhanced Chemical Vapor Deposition (PECVD). The deposition temperature of the a-SiC:H/a-Si:H layers is lower than 250 °C and therefore it allows the use of different kind of substrates including glass, one of the most used materials for the fabrication of microfluidic devices. Furthermore, the glass substrate hosting the photosensors can be used as an active mechanical support for a microfluidic network made from other materials, including polymers, which are fabricated at lower temperatures. Finally, a-Si:H photosensors present excellent optoelectronic characteristic such as high quantum efficiency (from 60% to 90% in the visible range) and low dark current (around 10^−10^ A/cm^2^ at small reverse voltage) [11].

These reasons make a-Si:H photosensors ideal candidates for LoC applications and in Point-of-Care (PoC) devices. However, thermal treatment of the analyte [12,13] or large temperature variations can affect the correct operation of the photodiode in detecting low-level radiation signals [14]. Indeed, under reverse bias conditions, an increase/decrease of the operating temperature leads to a variation of the sensor current [15], due to the exponential dependence on the temperature of the diode saturation current. These changes are not discernible from changes due to the light incident on the photosensor, and lead to a worse limit of detection (LOD).

In order to reduce these effects, this work proposes to use an a-SiC:H/a-Si:H differential photosensor [16]. Two identical amorphous silicon/amorphous silicon carbide heterojunctions are properly connected to measure the difference between the currents of the two diodes. In the proposed structure, one diode is light-shielded and therefore its current depends only on temperature, while the current of the other diode depends on temperature and light. Under these conditions, the current induced by temperature is the common signal, while photocurrent is the differential signal.

The parameter expressing the performance of a differential circuit is the common mode rejection ratio (CMRR). For our device, this parameter is defined as follows:

(1) $$\text{CMRR}=\frac{\Delta I_{1}+\Delta I_{2}}{2\cdot \Delta I_{\text{DIF}}}$$

where ∆*I*_1_ and ∆*I*_2_ are the current variations of the two diodes and ∆*I*_DIF_ is the variation of the differential current induced by a temperature variation ∆*T*.

The paper is organized as follows: Section 2 presents the device structure and the related fabrication steps, Section 3 reports the optoelectronic characterization of the individual junctions and of the differential current at room temperature, Section 4 experimentally describes the efficacy of our device in rejecting the temperature common mode signals, Section 5 presents the conclusions.

## 2. Device Structure and Fabrication

The proposed differential device is the series connection of two amorphous silicon sensors grown by Plasma Enhanced Chemical Vapor Deposition (PECVD) on a 5 × 5 cm^2^ glass substrate. Each sensor is a p-doped a-SiC:H/intrinsic a-Si:H/n-doped a-Si:H stacked structure.

**Figure 1:**
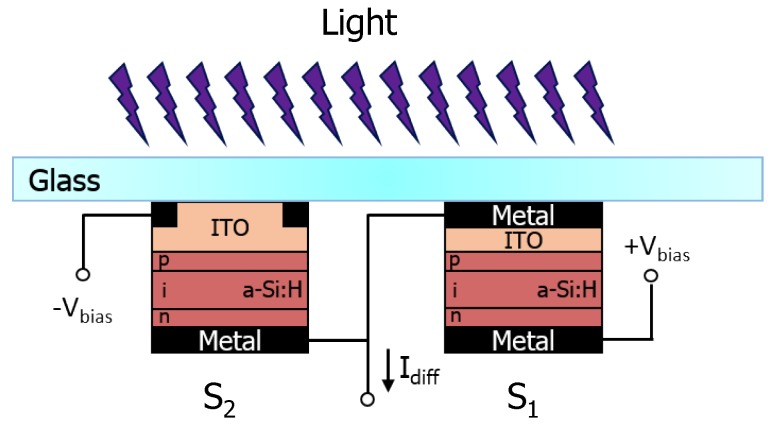
Device cross-section. S_1_ is the ligth-shielded junction and therefore it is sensitive only to temperature, while diode S_2_ is sensitive to both temperature and light.

The device structure and a schematic view of its electrical connection are depicted in Figure 1. The two p-i-n sensors differ only in the electrical bottom contact: in the sensor S1, the bottom contact is a stack of a metal and a indium tin oxide (ITO) transparent layer, while in the sensor S2 it is constituted by only the ITO layer. This configuration makes both diodes sensitive to temperature and solely S2 sensitive to light. Indeed, the two diodes are thermally coupled, and experience the same temperature, while only one is exposed to the radiation. In these conditions, temperature and light can be considered as the common and differential mode signals, respectively.

**Figure 2:**
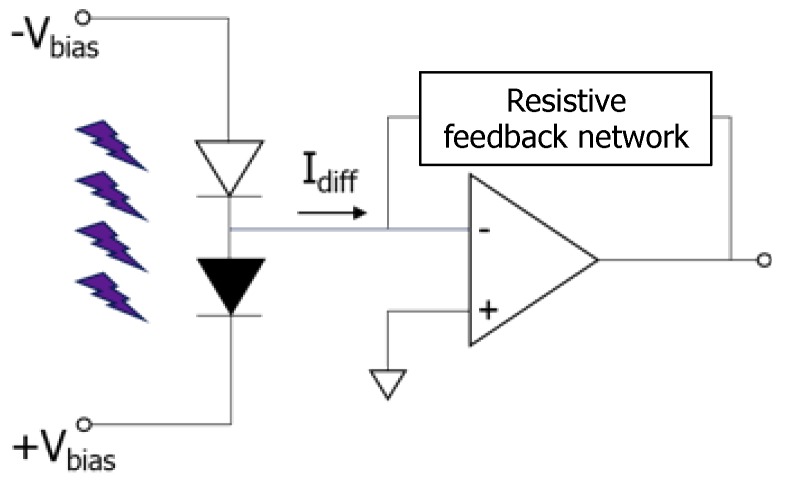
Schematic of the electrical connections of the differential structure. The two diodes work at reverse bias. A current to voltage converter circuit reads the differential current.

The device has three electrodes: two of them bias the sensors while the third one connects in series the two diodes and provides as output signal the difference between the diode currents. The measurement of the differential current is performed connecting the common electrode to a transimpedance amplifier as schematically shown in Figure 2.

**Figure 3:**
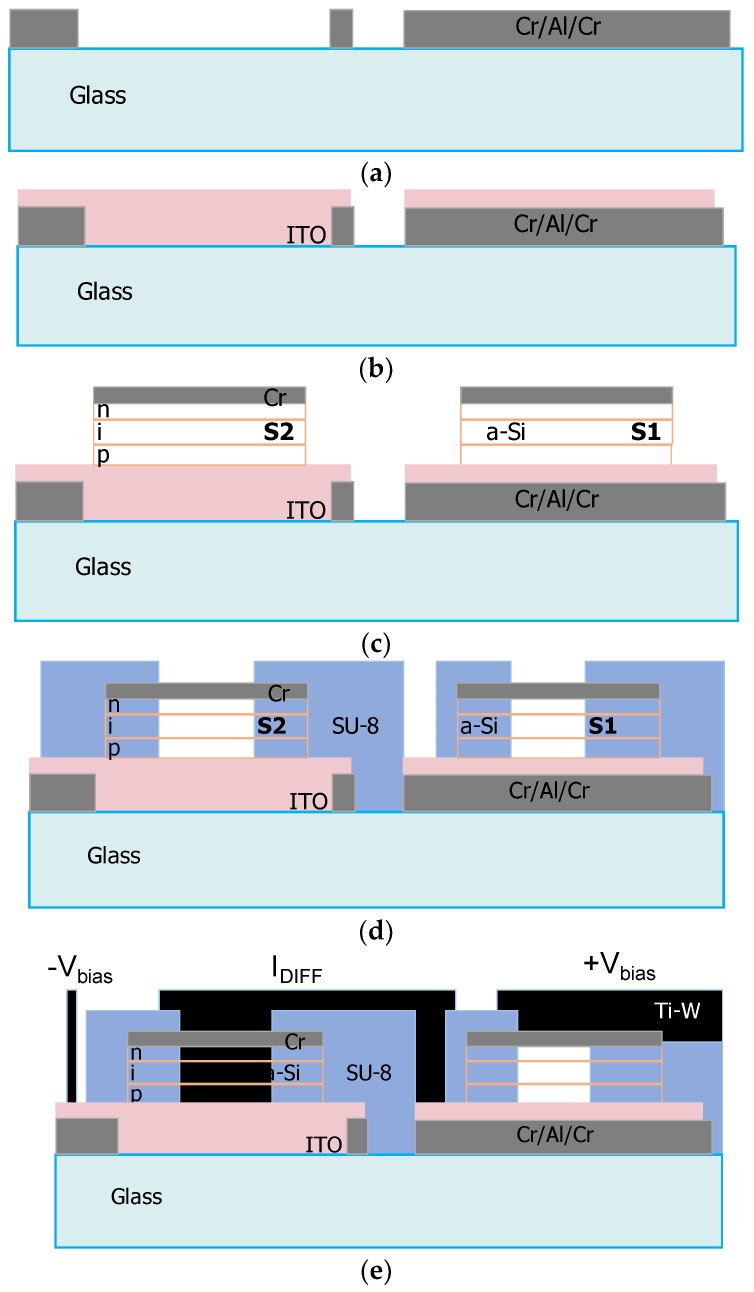
Fabrication flow of the differential device (see text for details), showing the deposition and patterning of the: (**a**) metal bottom contact; (**b**) Indium Tin Oxide (ITO) layer; (**c**) a-Si:H diodes; (**d**) SU-8 insulation layer; (**e**) top electrode.

**Figure 4:**
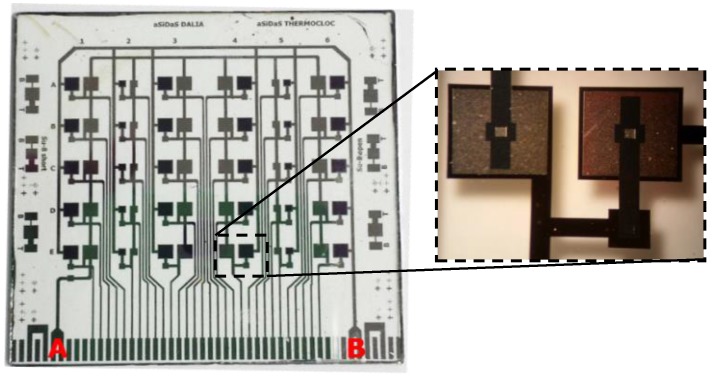
Picture of the 5 × 6 devices array on 5 × 5 cm^2^ glass substrate. A and B indicate the electrical contacts to the positive and negative bias voltage respectively. The photo on the right is a microscopic view of a single device.

**Figure 5:**
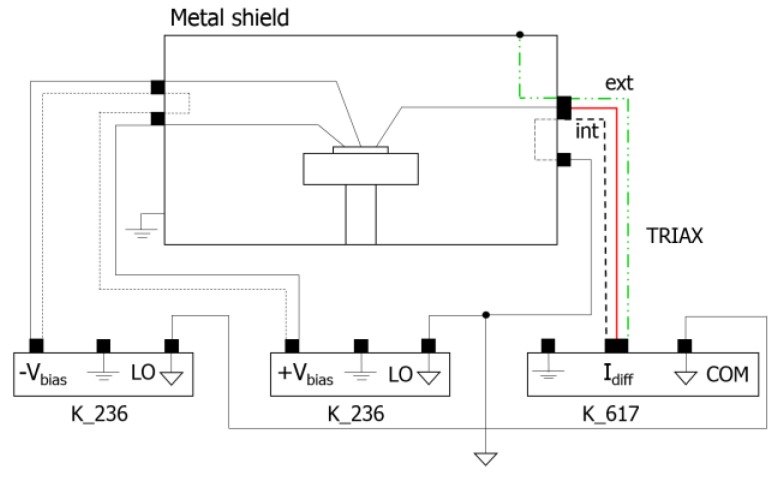
Scheme of the setup used for the measurement of the differential current. The metal box minimizes the electromagnetic noise.

**Figure 6:**
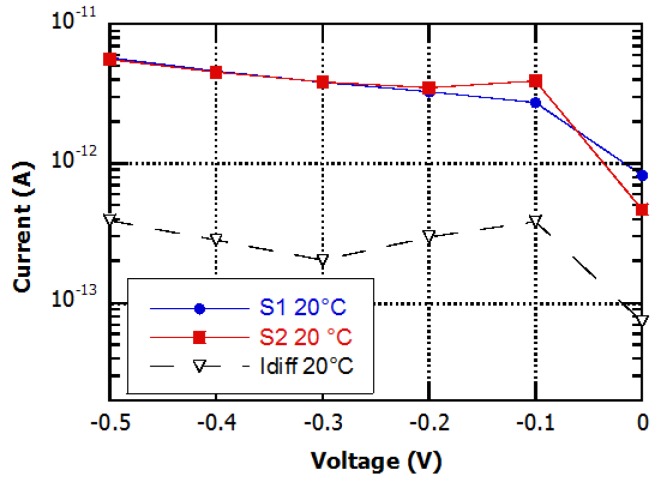
Current-voltage curves of the single diodes of the device and of the differential current measured in dark conditions at room temperature.

The device fabrication foresees a five mask process for the definition of the bottom electrodes (two masks), mesa of the a-Si:H sensors, via holes through the insulation layer and patterning of the top electrode. Taking into account the potential application of the proposed system in multiplex on-chip detection, the photolithographic masks have been designed as an array of symmetrical differential photodiodes. The fabrication process has been implemented with the following steps (see Figure 3):

(1) Vacuum evaporation of a 30-150-30 nm-thick Cr/Al/Cr metal layers and their patterning with mask #1 for the bottom contact of the light-shielded diodes (S1), its connection to the output electrodes and the polarization line of the light sensitive sensors (see Figure 3a);
(2) Deposition by magnetron sputtering of a 200 nm ITO layer and its etching in argon plasma, using mask #2, to define the transparent bottom contact of the photosensors (S2) (see Figure 3b);
(3) Deposition by PECVD of the p-i-n structure followed by a 50 nm-thick vacuum evaporated chromium top electrode layer and patterning of the device structure through mask #3 by wet etching for the metal layer and reactive ion etching for the a-SiC:H/a-Si:H films (see Figure 3c);
(4) Deposition of a 5 µm-thick insulation layer (SU-8 3005 from MicroChem, Westborough, MA, USA), definition of the via holes over the diodes through mask #4 (see Figure 3d);
(5) Sputtering of a titanium/tungsten (Ti-W) alloy layer, and patterning with mask #5 for the definition of the two-diode series connection and the common bias line of the light shielded sensors (see Figure 3e);
(6) Deposition of a protective 5 µm-thick passivation layer over the glass substrate.

The array is composed of 30 differential photodiodes: 20 of them have an 1800 × 1800 µm^2^ active area and the other 10 have an 800 × 800 µm^2^ active area. An example of the final array of devices is shown in Figure 4, where a microscopic picture of a single device is also reported. The two diodes are reverse biased by applying a voltage at the contacts A (+V_bias_, common polarization line of light-shield sensors) and B (−V_bias_, common polarization line of light sensitive sensors).

## 3. Device Characterization

Fabricated devices have been electrically and optically characterized at room temperature measuring the current-voltage (I-V) characteristics in dark conditions and the quantum efficiency (QE) curves in the wavelength range 400–700 nm. Reported results refer to one of the 1800 × 1800 µm^2^ active area device. From the experimental electrical characterization performed on the smaller sensors, we found a very good agreement between the ratio of the measured currents and the ratio of the device active area. Furthermore, a standard variation of about 6% has been inferred from current measurements carried out on 10 differential structures.

### 3.1. I-V Characterizations

At first, we measured the I-V curves of the differential structure with an experimental set-up designed to avoid electrical crosstalk between diodes [17]. To minimize the electromagnetic noise the measurements were performed in a Faraday cage. In practical LoC applications, the reduction of noise can be attained including the system in a small metal case as the one reported in [18].

Subsequently, we measured the differential current. The experimental setup used for this measurement is reported in Figure 5: two 236 Source Measure Units (Keithley Instruments, Inc., Cleveland, OH, USA) bias the two diodes of the device, while a 617 Electrometer reads the differential current using a transimpedance configuration.

Measurements have been performed in dark condition, at room temperature, on several devices. Typical results are shown in Figure 6. We found that, at small reverse bias voltage (between 0.1 and 0.5 V), the currents of the single diodes are close to 4 × 10^−12^ A, while the differential current is about one order of magnitude lower.

### 3.2. Quantum Efficiency Measurements

As it is well known, this measurement determines the sensor photoresponse as a function of wavelength. For our differential structure, this characterization is particularly important because it measures also the light-shield efficacy of the bottom metal. Indeed, the diode S1 has to be blind to illumination radiation and its current has to be affected only by temperature.

**Figure 7:**
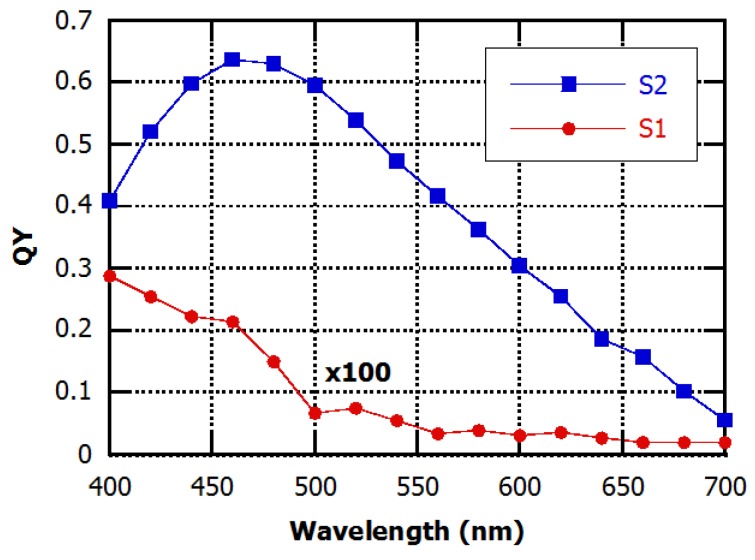
Quantum efficiency characteristics of the two diodes of the device at V_bias_ = 0 V and at room temperature.

In order to avoid cross talk issues, all the quantum efficiency curves have been measured at zero bias voltage. Figure 7 shows the quantum yield curves of the two single diodes. The QE of the temperature sensor S1 is much lower than the QE of the light sensitive sensor and therefore, to make it visible in Figure 7, it has been multiplied by 100. The different qualitative behavior of the two diodes and in particular of the increase of the QE signal of S1 with decreasing wavelength can be ascribed to the low reliability of the QE measurement at very low photocurrent levels. This occurs in the light-shielded sensor S1 due to its limited light sensitivity and to the low number of photon emitted by the halogen lamp used in the experimental set-up at low wavelengths. Nevertheless, the very low quantum yield (QY) of this sensor demonstrates the effectiveness of the metal shield to achieve optimal insulation from light radiation.

## 4. Results and Discussion

**Figure 8:**
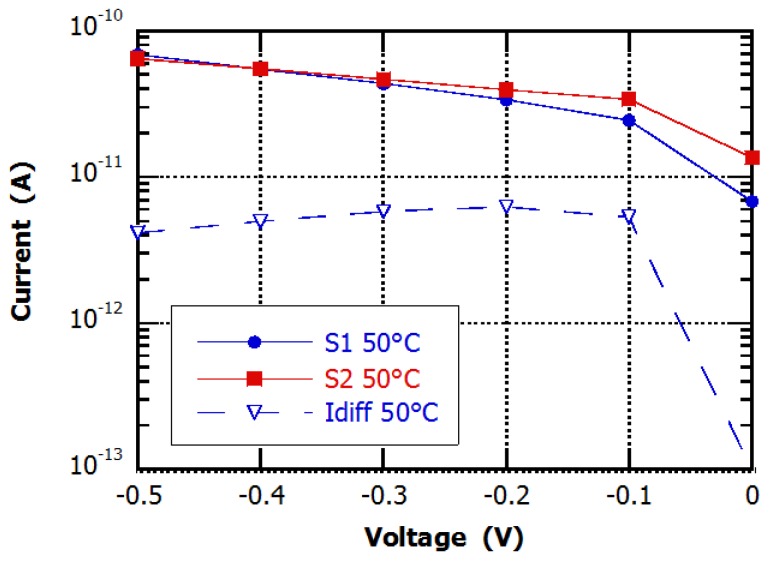
Current-voltage curves measured in dark conditions at 50 °C.

This part shows the suitability of the proposed device in reducing the temperature common mode signals and in detecting low-level radiation signal in a not-controlled temperature environment. To calculate the CMRR value of the device we measured the differential current and the single diode currents in reverse bias conditions (−0.5 to 0 V) at 50 °C, heating the sample with a metal chuck. a thermocouple placed on the glass hosting the photodiodes monitored the device temperature. Comparing results reported in Figure 8 with those obtained at 20 °C (see Figure 6), we note that, for each temperature, the differential structure is able to reject the current increase induced by temperature, Indeed, the differential current is about one order of magnitude lower than the current of each single diode. Applying Equation (1), we found CMMR values ranging from 15 to 24 dB. Ideally, the CMRR is infinite for perfectly matched structures. A limited value of CMRR is due to mismatches between the two diodes of the device structure.

In PoC applications, the environment temperature is usually not controlled/monitored and can have large variations from place to place. It is therefore important to verify the efficacy of proposed device to diminish the temperature effects when low-level signals have to be detected. In this case the LOD of the system is also related to the error in the measure of the light intensity induced by a temperature variation. Indeed under constant illumination condition, a current variation due to a temperature change adds to the photocurrent *I*_ph_ (defined as the difference between the total current and the current measured in dark condition). In particular, the LOD, defined as the minimum illumination intensity that can be correctly measured, is equal to three times the current variation induced by temperature.

**Figure 9:**
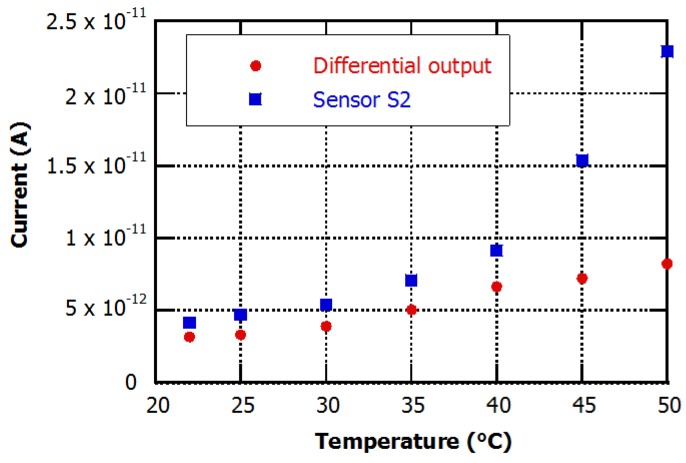
Experimental currents flowing through the light sensitive diode and at the differential output of the device under 10 pW of constant white light illumination.

Figure 9 reports the currents measured on the photosensor S2 and at the differential output of the device, in reverse bias (−0.1 V), at different temperatures and under 10 pW white light exposure, which induces in the S2 diode a photocurrent (*I*_ph_) value of 2.5 pA at room temperature (23 °C).

In previous works [18,19], we have found that the photocurrents induced in a single junction a-Si:H photosensors by actual chemi/bio-luminescent signals are in the order of few-tens of pA. Therefore, the value of 2.5 pA chosen in our experiment represents a signal induced by a number of molecules very close to be realistically detected in a lab-on-chip application.

The total current of diode S2 ranged from 4 pA at room temperature (23 °C) to 23 pA at 50 °C, with an exponential growth that matches the theoretical current-voltage relation of a photodiode [20]:

*I* = *I*_S_(e^−(q*V*d/k*T*)^ − 1) + *I*_ph_ (2)

where *I*_S_ is the reverse saturation current, q the electron charge, *V*_d_ the diode voltage, k the Boltzmann constant and *T* the absolute temperature. In particular, the value of the measured current at 50 °C corresponds to a light intensity equal to 90 pW, 10 times greater than the true value.

On the other hand, the differential current of the device varied from 3 pA at room temperature to 8.2 pA at 50 °C. The measured current at 50 °C corresponds to a light intensity equal to 30 pW, three times greater than the true value.

Furthermore, assuming an average responsivity of 250 mA·W^−1^, and taking into account the current variations due to temperature in our experiment, it can be inferred that the LOD is around 230 and 60 pW for the single diode and for the differential structure, respectively.

These results demonstrate that the proposed device provides an improvement of the performance with respect to the single diode structure in the specified applications. Better rejection factors with improved LOD can be obtained with higher diode matching which depends critically on the device fabrication processes used.

## 5. Conclusions

We have presented a device able to reduce the influence of temperature on detection of light in lab-on-chip and point-of care systems. The device structure is the series connection of two a-SiC:H/a-Si:H p-type/intrinsic/n-type diodes: one blind, sensitive only to temperature, and the other sensitive to both temperature and light. In these conditions, current induced by temperature is the common signal, while photocurrent is the differential signal. We measured a common mode rejection ratio up to 24 dB, which means an improvement of the limit of detection in biomolecular analysis of about one order of magnitude with respect to the single diode.
